# Writing scientific articles like a native English speaker: concise writing for Portuguese speakers

**DOI:** 10.6061/clinics/2016(12)01

**Published:** 2016-12

**Authors:** Mariel A Marlow

**Affiliations:** Centers for Disease Control and Prevention, Atlanta, GA, USA

As post-doctoral fellow in 2013, I published an editorial based on my experience as a translator, called “Writing scientific articles like a native English speaker: top ten tips for Portuguese speakers” (available at http://dx.doi.org/10.6061/clinics/2014(03)(01) 1. It focused on the repeated “simple” mistakes I observed while revising manuscripts of Brazilian friends and colleagues. I did not predict the editorial would receive as much attention as it did on social media. It speaks to Brazilian researchers’ quest for representation on the international scientific platform and the ever-growing number of Brazilian publications in international journals.

When I finished my post-doctoral fellowship, I left academia for government, starting a position as an Epidemic Intelligence Service (EIS) officer at the Centers for Disease Control and Prevention. Over the course of the two-year program, EIS officers learn applied public health by investigating outbreaks, making public health recommendations, and communicating public health issues to the public. The latter experience greatly changed my writing style. In the first month, I took a training called “Plain Language” developed by the National Institutes of Health (NIH). After applying the training to my communications as a public health official, I recognized my academic training prepared me to communicate my *scientific* findings to a *scientific* audience. Now my audience is the scientific community *and* the public. I was suddenly back on level one at a game I thought I had tirelessly conquered. Nevertheless, as with any skill, becoming a good writer is a matter of practice and persistence.

Here, I pass on the lessons I learned about plain language and concise writing. In a world of countless 24-hour news outlets, clearly communicating our findings so they are understood by the public is the first step to prevent the rapid spread of misinformation. I am not saying your Nature paper needs to read at a primary school level. You cannot avoid scientific terms and complex mechanisms, but you can make it easier for a reader to immediately identify your main point and follow your rationale. I selected the lessons below for their relevance to native Portuguese speakers from two sources. These are just a subset and I encourage you to read both sources in their entirety. First is the NIH Plain Language Training, which is publicly available at https://plainlanguage.nih.gov/CBTs/PlainLanguage/login.asp 2. The next is the book titled “On Writing Well: The Classic Guide to Writing Nonfiction” written by William Zinsser, a renowned journalist, editor, and professor 3. In his own words, “[s]cience, demystified, is just another nonfiction subject. Writing, demystified, is just another way for scientists to transmit what they know.” You are a nonfiction writer. Also, broadening your writing skills from manuscript writing to other forms of scientific communication will make you a better scientist and advocate.

## 1. TAKE THE EXTRA STEP TO USE ACTIVE VOICE

In the first editorial, I encouraged the use of passive voice but also included the disclaimer that passive voice is a contentious topic and generally considered bad writing. There were two reasons for my initial support of passive voice. First, passive voice sounds more classic, just like an English accent. We take comfort in sounding more credible, i.e., more academic. But what we are actually doing is isolating ourselves from the wider audience by being boring and confusing it. Second, passive voice is commonly used in Portuguese texts and direct translations are easier. But an easier translation does not mean a better translation. Translators also consider a direct translation more accurate, faithful and authentic. The Portuguese language is not a direct, concise language. If a direct, concise scientific article is our objective, then we will need to make adjustments in the English translation.

I am now a firm supporter of active voice in scientific writing. Active voice means the subject of your sentence performs the action, with the subject coming before the verb. This results in a more direct tone that is easier to read for all audiences. As an added bonus, it will reduce your word count.

You have two options for achieving active voice. You can translate Portuguese passive voice to English passive voice and then change to active voice.

### Example 1

Portuguese: *Neste artigo foram apresentados os principais aspectos da escrita científica.*

English passive voice translation: *In this article the principal aspects of scientific writing were presented.*

This is an easy translation because all parts of the sentence are directly translated. You just need to slightly rearrange, but even Google Translate will do that for you. The subject is assumed; who is doing the presenting is not specified. This phrase in English is vague and wordy.

English active voice translation: *In this article, we present the principal aspects of scientific writing.*

This is more difficult to reach. If directly translating to English from the original phrase, you would need to change the subject from “assumed” to “we” and the verb from “to be” plus the past participle to an action verb. This requires a more advanced grasp of the English language – or does it? Active voice is likely how you first learned English. It is also understood better by those with basic English language skills. Writing in active voice does not require more advanced English, it just takes more effort.

Or you can write in active voice in Portuguese before translating to English.

### Example 2

Portuguese passive voice: *Dois mil casos da doença foram relatados em cinco estados.*

Portuguese active voice: *Cinco estados relataram dois mil casos da doença.*

English translation (already in active voice): *Five states reported two thousand cases of the disease.*

Reread the example phrases. Which sounds clearer? Are you more comfortable using active voice in Portuguese or after translation to English?

## 2. START WITH ONE FACT (YOUR RESEARCH QUESTION) AND BUILD ON IT

For graduate students (*pós-graduação*), one of the first major writing experiences is a thesis or dissertation. This is in no way a concise document, unless you want your defense committee to think you spent your two or four years on the beach. Leaving this experience, you need to practice promoting your work beyond a defense committee. The rest of the world, especially those in the same field, does not want to know every fact you know about your research topic. Being concise helps you focus. Focused ideas are more easily understood.

The quickest way from Point A to Point B is a straight line. That is your goal. Point A is your research question. Point B is a reader understanding your results. To reach Point B you must build the story for them. Readers will keep relating back to what they understood was the research question – Point A. Your research question must be clear. If readers cannot directly relate what you write to Point A, they may get lost. Make an outline of each major point you want to make. If you can’t relate it back to Point A, then it may fail to support your answer to Point A. William Zinsser equates this fact-building-upon-fact method to an upside-down pyramid. This is a great analogy especially when communicating your research beyond your manuscript. You start with one fact that a reader must know and then lead them up the pyramid. You build on what you told them until you reach the broader application of your results.

## 3. REMOVE UNNECESSARY NOUNS AND ADJECTIVES

Before translating to English, identify phrases that could be expressed with a single word.

### Examples

Avoid redundant expressions.

### Examples

Avoid extraneous information.

### Example

*Patient required intubation and mechanical ventilation.* A patient who requires mechanical ventilation would have to be intubated. The fact that the patient was intubated is therefore unnecessary information. *Patient required mechanical ventilation.*

After translating to English, you can also apply tips from the first editorial, such as avoid starting sentences with “It is” (or “There are”), place adjectives in front of nouns when possible (adjective-noun rather than noun-“of”-adjective), and remove unnecessary “that”.

## 4. REMOVE UNNECESSARY VERBS OR VERBS HIDDEN AS NOUNS

Before translating to English, highlight the following verbs in your document: *fazer* (to make or do), *ter* (to have), *realizar* (to perform), *fornecer* (to provide), *produzir* (to produce), *dar* (to give) and *conduzir* (to conduct). Often these verbs precede nouns that can be transformed into the verb. This also helps change a phrase into active voice.

### Examples

*Realizar entrevistas* can be reworded as *entrevistar*:

Depois do consentimento, realizamos entrevistas com os responsáveis das crianças.

Depois do consentimento, entrevistamos os responsáveis das crianças.

### Other common verb phrases

Fazer uma análise – analisar.

Conduzir um exame – examinar.

*Realizar um estudo – estudar*.

Next, highlight nouns ending with *-ção* or *-são* (in English -ion). These nouns are often verbs transformed into nouns and can be reverted. The verbs *estar* (to be) or *dar* (to give) usually precede them. Some of the most common in scientific writing are *introdução*, *conclusão*, *observação*, and *interpretação*.

### Examples

If the verb does not exist in Portuguese, check if one exists in English after translation.

## 5. NOW GO BACK AND CUT, CUT, CUT, AND THEN CUT SOME MORE

Cut not only unnecessary words, but also unnecessary content. Do your readers need to know that the disease was first discovered in 1967? Do they *really*? Again, this all goes back to the importance of having a research question. If the information is not directly relevant to why you pursued the research question, how you approached your research question, or how your results answer the research question, consider removing it. Are you writing a review demonstrating the lack of research on the disease? Then yes, the discovery in 1967 may be important to include. Are you writing a manuscript on your results from a case-control study conducted in 2015? Then probably not.

Word counts are your friend, not your enemy. They don’t keep you from including important information in your abstract; they do prevent you from including information you think is important that can wait for the reader to read the article. They don’t prevent you from comparing your study with all related studies in your discussion; they do prevent you from losing your reader’s attention with extraneous information. They don’t prevent you from expanding on how your results apply to a larger context; they do help prevent you from expanding on topics that are not relevant to your conclusions.

As you tighten-up your word count, you may realize you’ve said the same thing more than once, in different ways – say it just once! Then after you are done, put it away for a day or more. And then, with fresh eyes, revise some more.

Your goal is to say it in as few words as possible. You might even find there is an austere elegance to concise writing you never noticed before.

## Figures and Tables

**Table:** 

Longer phrase	Concise
*Até o momento*	*até*
*Por meio de*	*por*
*no caso de*	*se*
*Fora da realidade*	*impossível*

**Table:** 

Redundant	Concise
*Metades iguais*	*Metades*
*Exportar para fora/Importar para dentro*	*Exportar/importar*
*Conclusão final*	*Conclusão*
*Criar novo*	*Criar*
*Países do mundo*	*Países*
*Manter o mesmo*	*Manter*
*Repetir de novo*	*Repetir*
*Planos para o futuro*	*Planos*
*Introduzir dentro*	*Introduzir*

**Table:** 

Phrase	Verb
*Estar/dar prevenção*	*prevenir or evitar*
*Dar proteção*	*proteger*
*Dar informação*	*informar*
*Chegar a uma conclusão*	*concluir*
